# Trends in epidemiology: the role of denominator fluctuation in population based estimates

**DOI:** 10.3934/publichealth.2021040

**Published:** 2021-07-08

**Authors:** Emanuele Amodio, Maurizio Zarcone, Alessandra Casuccio, Francesco Vitale

**Affiliations:** University of Palermo, Department of Health Promotion Sciences, Maternal and Infant Care, Internal Medicine and Medical Specialties “G. D'Alessandro”. Via Del Vespro 133, Palermo, Italy

**Keywords:** population estimates, censal data, public health

## Abstract

**Background:**

Population estimates are of paramount importance for calculating occurrence and association measures although they can be affected by problems of accuracy and completeness. This study has performed a simulation of the impact of Italian population size variability on incidence rates.

**Methods:**

Data have been obtained by the Italian National Institute of Statistics. For each year expected cases were calculated at increasing fixed rates (up to 1,000/100,000) and were considered constant in the “following year”, calculating statistical differences (P < 0.05).

**Results:**

In Italy and in other regions, statistically significant higher RRs were found in 2012 vs. 2011 whereas statistically significant lower RRs were found in 2013 vs. 2012 and in 2014 vs. 2013.

**Contribution:**

The simulation confirms that significant differences due to population fluctuation could be found between consecutive years when investigating diseases with medium-high rates. Researchers should be encouraged to implement actions for reducing the risk of biased population denominators.

## 1. Introduction

Population estimates are of paramount importance in epidemiology since they represent denominators of occurrence measures and can be involved in calculation of risk rate ratios. For this reason, almost all high income countries in the world have developed national open-access databases that can be freely used by all scientists for obtaining population data stratified by gender, age and area level (state, region, province, city etc) [1]. In Europe these data are collected on an annual basis by the European Commission and include data on general population, live births and deaths occurred during the reference year and, usually, the total number of immigrated and emigrated subjects during the course of the year [2].

All these datasets are daily consulted by researchers for calculating incidence rates and prevalence of diseases as well as trends over time, comparing different areas or sub-populations with different demographic patterns.

In a large majority of studies that use public demographic data, scientists have to consider the role of data accuracy and completeness, since variations in these measures could lead to biased estimates and, consequently, to imprecise or erroneous epidemiological conclusions. In particular, in the last decades there has been an increasing interest in this active area of research with analysts trying to improve denominator estimates, evaluating the importance of this uncertainty and developing methods to handle the problem. In this sense, in Italy some criticisms in population registration process seem to be occurred in the years following the 2011 national Census [3]. There is the suspicion that largely fluctuating population sizes, when numerator (“cases of diseases”) is stable over time, could statistically significantly affect differences between occurrence rates.

According to these perplexities, by using data collected by the Italian National Institute of Statistics [4], this study has carried out a simulation of the impact of population size variability on incidence rates between couple of consecutive years with the same number of incident cases.

## 2. Methods

All data used in this study have been downloaded by the demo.istat web section of the ISTAT [5], that is an Italian public research organization producing official statistics in the service of citizens and policy-makers. In particular data from 1^st^ January to 2002 to 1^st^ January 2015 were considered with stratification by age, sex and Italian region of residency. These data are based on a continuous observation of the municipalities existing and considering the territorial evolution through time (birth and death of municipalities by aggregation, disaggregation, transfer from a province or region to another).

For years from 2002 to 2011 inter-censal resident population estimates, reconstructed backward by the ISTAT after the release of the Legal Population of Municipalities at the 2011 Population Census, were also used [5].

For the simulation analysis, years from 2011 to 2014 were considered. For each year (denoted as “index year”), expected cases were calculated at increasing arbitrarily fixed incidence rates (1/100,000; 10/100,000; 100/100,000 and 1,000/100,000), and the number of cases found in the “index year” (e.g. 2011) weas used as numerator for the “following year” (e.g. 2012), thus hypothesizing a constant number of cases between consecutive years. Arbitrarily fixed incidence of each “index year” was then compared with incidence in the “following year” in order to evaluate statistically significant differences, on a regional and national basis. For each comparison, relative risks (RRs) were calculated considering the “index year” as reference. P-value for testing the hypothesis that the true RR is equal to 1 and 95% confidence intervals were computed according to Mantel-Haenszel method proposed by Rothman and Greendland [6].

All calculations have been made by using R software version 3.5.2 [7].

## 3. Results

**Figure 1.**
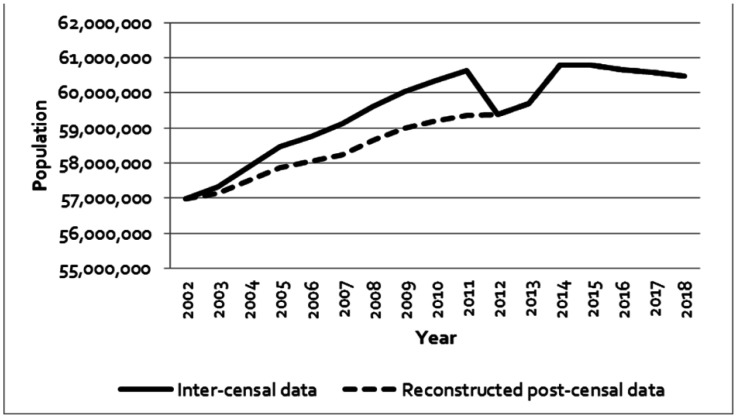
Annual Italian population size from 2002 to 2018 (2002–2011 reconstructed post-censal series in dashed line).

Figure 1 depicts trends of annual Italian population from 2002 to 2018. For years 2002 to 2011 data included both inter-censal and reconstructed post-censal series. As shown, in 2011 data from Census reported 1,261,752 less residents than those calculated in inter-censal series (59,433,744 vs. 60,785,753, respectively). From 2013 to 2014 a significant increase in Italian population was recorded (from 59,685,227 in 2013 to 60,782,668 in 2014).

**Figure 2.**
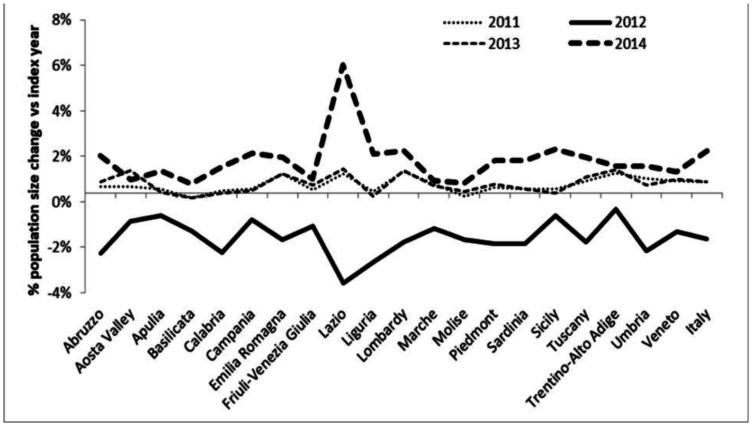
Population size percent change in Italian Regions with respect to the previous year (“index year”).

In Figure 2 the population percent change during a four year period (2011 to 2014) was reported for all Italian regions. In 2012 a decrease in population size was registered in all Italian regions (from −4.0% for Lazio to −0.73% for Trentino-Alto Adige) whereas in 2014 all regions recorded an increase in population size ranging from +5.6% (Lazio) to +0.38% (Basilicata).

In Table 1, trends of annual percentage changes stratified by age and sex have been reported. A particular decrease was evident in 2012, in both males and females, for 0–19 (−2.51% and −2.24%, respectively) and 20–39 (−5.22% and 4.33%, respectively) years old age groups. Otherwise subjects aged 80 years or more had a substantial increase in 2014 (+4.03% in males and +2.77% in females).

**Table 1.** Annual population size percent change stratified by age and sex (vs “index year”).

	Males				Females			
	2011	2012	2013	2014	2011	2012	2013	2014
0–19	0.03%	−2.51%	0.04%	1.43%	0.01%	−2.24%	−0.09%	1.03%
20–39	−1.22%	−5.22%	−1.32%	0.45%	−1.10%	−4.33%	−1.50%	−0.14%
40–59	1.22%	−1.36%	1.69%	3.40%	1.49%	−0.53%	1.57%	3.13%
60–79	1.04%	−0.66%	1.19%	2.25%	0.52%	−0.91%	0.72%	1.60%
80–100	4.74%	1.96%	3.58%	4.03%	3.47%	0.77%	2.31%	2.77%
All age groups	0.43%	−2.33%	0.57%	2.06%	0.52%	−1.75%	0.42%	1.63%

**Table 2.** Relative risks (RRs) and statistical significance (as apices) of detecting incidence rates different from that arbitrarily fixed in “index year”.

	2012 vs. 2011	2013 vs. 2012	2014 vs. 2013
Abruzzo	1.0275^d^	0.9954	0.9839
Aosta Valley	1.0127	0.9904	0.9942
Apulia	1.0102	0.9998	0.9904
Basilicata	1.0172	1.0024	0.9962
Calabria	1.0271^d^	1.0001	0.9887
Campania	1.0121^d^	0.9991	0.9829^d^
Emilia Romagna	1.021^d^	0.9917	0.9845^d^
Friuli-Venezia Giulia	1.0148	0.9967	0.9939
Lazio	1.0416^c,d^	0.9897	0.9467^c,d^
Liguria	1.0315^d^	1.0014	0.9832
Lombardy	1.0224^d^	0.9904^d^	0.9821^d^
Marche	1.016	0.9971	0.9949
Molise	1.0212	0.9994	0.9956
Piedmont	1.0229^d^	0.9963	0.9859d
Sardinia	1.0229^d^	0.9985	0.9859
Sicily	1.0102	0.9999	0.9814^d^
Tuscany	1.0224^d^	0.9932	0.9846^d^
Trentino-Alto Adige	1.0073	0.9900	0.9886
Umbria	1.0263	0.9966	0.9883
Veneto	1.0173^d^	0.9942	0.9909
Italy	1.0207^c,d^	0.9951^d^	0.9819^c,d^

Note: Statistically significant difference with “index year” fixed at incidence rate: (a) 1/100,000; (b) 10/100,000; (c) 100/100,000 and (d) 1,000/100,000.

Table 2 summarizes comparisons between each couple of years (“index year” as reference). Statistically significant higher RRs (ranging from 1.012 and 1.0416) were found in 2012 vs. 2011 in Italy and in other 11 regions for “index year” incidence fixed at 1,000/100,000 cases per year and in Italy and Lazio for “index year” incidence fixed at 100/100,000 cases per year. In 2013 vs. 2012 Italy and Lazio showed statistically significant lower RRs for “index year” incidence rates fixed at 1,000/100,000 cases per year.

In 2014 vs. 2013 statistically significant lower RRs (ranging from 0.9467 and 0.9846) were found in Italy and in other 6 regions for “index year” incidence fixed at 1,000/100,000 cases per year and in Italy and Lazio for “index year” incidence fixed at 100/100,000 cases per year.

## 4. Discussion and conclusions

The present paper intended to evaluate a methodological aspect that seems to be commonly neglected during epidemiological investigations on general population including those carried out by cancer and mortality registries, epidemiological surveillance systems and health agency reports. The question under discussion is if population data, used as denominator in epidemiological studies, can represent a source of bias and how large can be the weight of this bias when present. Some authors suggest that errors in denominator terms can have a non-trivial impact on the results. In particular, in suburbanized areas population size can vary up to a 20%. When discrepancies of this magnitude occur an estimated incidence rate ratio can be 1.5 times the true ratio [8]. Other authors have estimated that over 10-year periods starting at the previous census, a substantial amount of error may accumulate ranging between as low as 10% and as high as 80% within any age/sex five-year age group [9],[10].

For investigating this issue, a simulation on Italian data collected by a national research organization (ISTAT) has been performed. The hypothesis was that a relatively large variation of population size between years with a constant number of cases could determine statistically significant differences in incidence rates. These differences are of great interest since they could represent the basis for implementing public health control strategies that could be avoided with unbiased population estimations. The simulation seems to confirm that significant differences due to population size could be found between years, both at a regional or national level, when investigating diseases with medium-high incidence rates (usually >100/100,000). This finding is quite expected since as the number of cases or events (numerator) and the study population (denominator) increase the relative width of the confidence interval becomes narrower [11]. In particular in 2012, the important decrease in population size at fixed number of cases determined a consequent increase in incidence rates with higher RRs, that were statistically significant at a national level and also in some regions. Otherwise in 2014 the increase in population size lowered incidence rates with protective RRs with respect to 2013.

It should be noted that the ISTAT have clarified that, between 2011 and 2014, the variability in population size could be at least in part attributable to some discrepancies between 2011 Census and Legal Population of Municipalities data. In this period, these discrepancies have determined 1,243,957 corrections (1,610,058 in plus and 366,101 in minus) of which about 85% was performed in 2013 [3].

In the pre-census period, some Italian authors have found that using inter-censal population as denominator for the year 2002–2011 produces a remarkable distortion of both temporal trend and geographical comparisons for cancer incidence rates, confirming some perplexities reported in this paper [12]. Some other authors have highlighted that particular race-specific estimates could prove far less reliable, with severe overestimates and underestimates of all racial groups in various counties nationwide [13].

Considering the reported problems in estimating population size and in absence of gold standard data, researchers should be encouraged to implement actions for reducing the risk of biased population denominators, especially for years where denominators are known to or have been declared to be fluctuating [14]–[17].

Ignoring the problem could be considered a possible solution when the numerator has a quit small incidence rates (<100/100,000 as we have simulated) or when variations in the size of the population are likely to be very small compared to the estimated effects. Otherwise, when one of the two previous situations is not satisfied, the international literature suggests several ways to try to take into account these problems. The first of these possibilities is to add to the statistical model a variable that allow the researchers to control for a proxy that is theoretically related to the size of the exposed population. A second possibility is to perform a mathematical estimation of the denominator by using some other data (for example interpolation by using other years data or moving means) or evaluating results by a sensitivity analysis [18] or other methods [19]. Finally, it can be also considered the possibility to deal with problem by study design as in the case of case-crossover design where population denominators are not used. More recently Jung et al have reported that, in some circumstances, new adjustment method dramatically enhances the statistical validity of global and local spatial autocorrelation statistics [20].

Of course, the present study has several limitations. First of all, it is a simulation and thus it could be not representative of events in the real life. As second point, demographic data could be more accurate than that assumed in this paper and thus, in this last case, calculated RRs would have estimated the true incidence rate ratios. Moreover, we have chosen to not add a further random noise to the numerator since our model considers that numerators (and, thus, the cases of diseases) are observed with a very high accuracy whereas when considering diseases with concerns of case-finding our model could be subjected to a type I error.

Finally, incidence rates were compared without adjustment for age and sex that could have an impact on estimates. In this sense, the large variability found in Italian population changes by age groups in different years seems to suggest that adjusted-rates could increase the statistical significant differences between estimates.

Despite these possible limits, this paper shows that in epidemiological studies the attention directed toward the ascertainment of accurate numerators can be not considered enough to warrant unbiased estimates when denominators are fluctuating and potentially inaccurate.
